# Cost-Effective Double-Layer Hydrogel Composites for Wound Dressing Applications

**DOI:** 10.3390/polym10030305

**Published:** 2018-03-12

**Authors:** Javad Tavakoli, Samaneh Mirzaei, Youhong Tang

**Affiliations:** 1 Centre for NanoScale Science and Technology, College of Science and Engineering, Flinders University, Tonsley, SA 5042, Australia; javad.tavakoli@flinders.edu.au; 2 The Medical Device Research Institute, College of Science and Engineering, Flinders University, Tonsley, SA 5042, Australia; 3 Stem Cell Technology Research Centre (STRC), 199777555 Tehran, Iran; smn.mirzaei@strc.ac.ir

**Keywords:** wound healing, double-layer hydrogel, mechanical properties, biocompatibility, adhesion

## Abstract

Although poly vinyl alcohol-poly acrylic acid (PVA-PAA) composites have been widely used for biomedical applications, their incorporation into double-layer assembled thin films has been limited because the interfacial binding materials negatively influence the water uptake capacity of PVA. To minimize the effect of interfacial binding, a simple method for the fabrication of a double-layered PVA-PAA hydrogel was introduced, and its biomedical properties were evaluated in this study. Our results revealed that the addition of PAA layers on the surface of PVA significantly increased the swelling properties. Compared to PVA, the equilibrium swelling ratio of the PVA-PAA hydrogel increased (*p* = 0.035) and its water vapour permeability significantly decreased (*p* = 0.04). Statistical analysis revealed that an increase in pH value from 7 to 10 as well as the addition of PAA at pH = 7 significantly increased the adhesion force (*p* < 0.04). The mechanical properties—including ultimate tensile strength, modulus, and elongation at break—remained approximately untouched compared to PVA. A significant increase in biocompatibility was found after day 7 (*p* = 0.016). A higher release rate for tetracycline was found at pH = 8 compared to neutral pH.

## 1. Introduction

Hydrogel-based wound dressings are well-known for promoting wound healing processes, while being able to maintain a moist environment and absorb wound exudates [1,2]. Their appropriate response to external actuation, their biocompatibility, and their adjustable swelling time and ratio have made them popular among other polymeric candidates [3,4,5]. Due to their inherent simplicity of fabrication, hydrogel wound dressings have been introduced in different forms of foam, film, membrane, and electrospun fibres [6,7,8,9]. However, different methods of fabrication directly influence the cost of production. The simpler the method (if it leads to an efficient product), the more attractive it is. 

Among different hydrogels, poly vinyl alcohol (PVA) has drawn much attention for wound dressing applications; its numerous remarkable biomedical properties have been described well [10,11,12]. PVA with a high degree of hydrolysis (>99%) provides a promising hydrophilic network that has been widely used for drug delivery purposes [13,14]. However, its application in hydrogel form is limited for transcutaneous wound dressings due to its poor adhesion property, neutral characteristic, relatively low water uptake, and highly crystalline regions that are impermeable to oxygen diffusion [15]. To improve the biomedical properties of PVA, attention has been paid to fabricating PVA-based hybrid hydrogels [16,17]. The addition of poly acrylic acid (PAA) to fabricate PVA-PAA hybrid films has been proposed as an opportunity to combine the enviro-sensitivity of PAA and the outstanding mechanical properties of PVA to fabricate a hybrid wound dressing with desirable characteristics [18,19]. With its distinctive biomedical properties, PAA can provide versatile structures to enhance the swelling, adhesion, diffusion, mechanical, and enviro-sensitivity properties of PVA wound dressings [20].

PVA-PAA composites have been widely used in biomedical applications [21]. Many studies have focused on the fabrication and measurement of the biomedical properties of PVA-PAA blends [22,23,24,25], but their incorporation into layer-by-layer assembled thin films has been limited because the interface-binding materials negatively influenced the water uptake capacity of PVA [26]. Borax complex, covalent bonding, blending, and hydrophobic interaction have been suggested to improve interfacial strength [27,28,29]. To minimize the effect of interface binding on the swelling properties of layer-assembled PVA, hydrogen bonding strategies have been suggested, although they clearly lack strong interface bindings [30]. Clay nanoparticles and polyaniline have been used as interface binding partners to assemble PVA-PAA multilayers [31,32,33]. Although the addition of interface partners has provided unique biomedical properties to the assembled hydrogel, delamination remains a concern. On the other hand, pre- and post- chemical treatments to enhance interfacial strength are usually expensive and time-consuming—features that discourage upscaling to commercialization.

This study proposes a simple method for the fabrication of a PVA-PAA double-layer structure to enhance the biomedical properties of the layered hydrogel. This novel and cost-effective approach for the fabrication of double-layered hydrogels has not been reported before. This method can improve the interface binding and has the potential to be used in the fabrication of multilayered hydrogels. The impact of PAA on the hydrophilic property of the PVA, the influence of PVA on the mechanical properties of PAA, and the evaluation of the biomedical properties of the assembled hydrogel are fully described.

## 2. Materials and Characterisations

The acrylic acid (AA) monomer (CAS Number: 79-10-7) was purchased from Sigma Aldrich (St. Louis, MO, USA) and was vacuum distilled at 5 mmHg at 30 °C to remove the inhibitor hydroquinone monomethyl ether. PVA (*M*_w_ =145,000; degree of hydrolysis = 99.9%), ethylene glycol dimethacrylate (EGDMA) as crosslinking agent, ammonium persulfate (AS), sodium bisulfide (SB), MTT (methylthiazolyldiphenyl-tetrazolium bromide) solution (TOX1), dimethyl sulfoxide (DMSO, 67-68-5), bovine serum (12203C), Dulbecco’s modified Eagle’s medium (DMEM, D5796), penicillin streptomycin (P4333), NaH_2_PO_4_, NaCl, NaHCO_3_, KCl, Na_2_HpO_4_, KH_2_PO_4_, phosphoric acid, and phosphate buffered saline (bio-performance certified, 0.2 μ-filtered) were obtained from Sigma Aldrich and were used with no further purification.

### 2.1. Hydrogel Preparation

**Figure 1:**
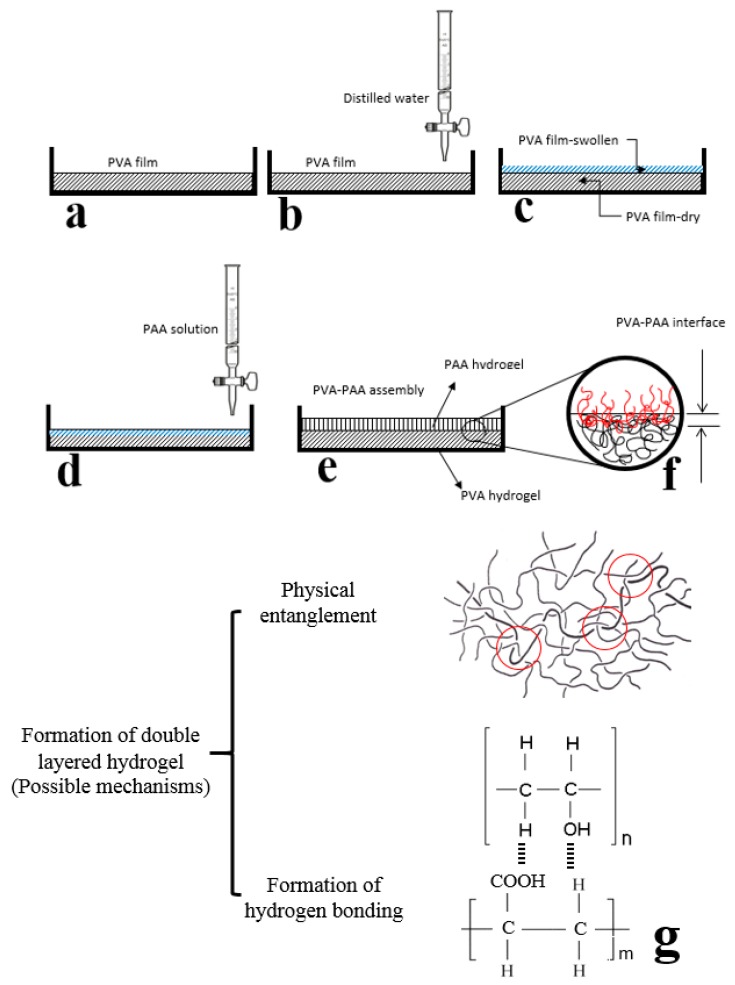
Schematic drawing of sample preparation steps. PAA: poly acrylic acid; PVA: poly vinyl alcohol. PVA films were prepared by the solvent casting method (**a**). A desired amount of distilled water was uniformly poured over the PVA film surface (**b**) for surface partial swelling (**c**) before addition of acrylic acid monomer (**d**) and polymerization process (**e**). The schematic drawing of PVA-PAA interface (**f**) and the possible mechanism for formation of double layered hydrogel at the interface (**g**) was shown at higher magnification.

PVA films were prepared by the solvent casting method. The solution (5% *w*/*w*), which was prepared by mixing of PVA powder and distilled water at 90 °C with constant stirring (1000 rpm) overnight in a sealed container, was poured into a Petri dish and placed at 70 °C to dry. Then, 0.1 mole acrylic acid (AA) monomer was mixed with 7 mL distilled water and 0.01 g EGDMA. In different containers, 0.06 and 0.05 mmol AS and SB, respectively, were mixed with 1 mL distilled water. A desired amount of distilled water (equal to approximately 10% by weight of PVA water uptake at equilibrium state) was uniformly poured over the previous PVA film surface and sealed at room temperature. After 30 min, distilled water was absorbed by the superficial layer of the PVA film and a distinct and thin rubbery layer was observed over the thick glassy layer. Then, the three solutions were mixed together, and the mixture was uniformly poured over the PVA rubbery layer using a shaker for 5 min. The crosslinking reaction of the AA mixture was allowed to proceed for 1 h at 50 °C. The sample preparation process is schematically shown in Figure 1.

### 2.2. Characterisation

To investigate PAA formation on the PVA surface during sample preparation as well as the possible interactions between PVA and tetracycline hydrochloride, Fourier Transform Infrared (FTIR) spectroscopy (Spectrum 400, PerkinElmer, Waltham, MA, USA) was performed using the transmittance mode. FTIR spectra were obtained in the range of wavenumbers from 4000 to 550 cm^−1^ during 64 scans, with 2 cm^−1^ resolution.

The swelling kinetics of PVA and PVA-PAA samples were characterised in water, and their sensitivity to pH change was measured in different buffer solutions by dissolving 0.35 g NaH_2_PO_4_, 0.68 g NaCl, 2.5 g NaHCO_3_, and 0.22 g KCl, 0.15 g Na_2_HPO_4_, 0.024 g KH_2_PO_4_ in 100 mL of distilled water, and the pH value was adjusted by using phosphoric acid and sodium hydroxide to 5, 7, and 10, respectively. For all samples and different swelling environments, the change in swelling ratio was measured at different time points until equilibrium using Equation (1):

(1) $$SR=\frac{M_{t}-M_{0}}{M_{0}}\times 100,$$

where *M*_t_ and *M*_0_ are sample weights at a time point and dry state, respectively. The average value (95% CI) of five measurements was reported. 

The hydrophilicity of samples was evaluated by using contact angle and water vapour permeability analysis. The contact angle was measured using the sessile drop method, where the image of 20 µL deionised water dropped on the surface was captured. The contact angle reported was the mean (95% CI) of five different spots in three PVA and PVA-PAA samples. Water vapour permeability of the samples was measured using a previously described method [6]. Briefly, circular samples (2 cm diameter) were attached on the opening of tubes using cyanoacrylate adhesive after the tubes were filled with 7 mL distilled water. All tubes were kept in a desiccator (humidity ≈ 75%) for 72 h and water vapour permeability was measured by Equation (2):

(2) $$water\;vapour\;permeability=\frac{\delta m}{A\times \delta t},$$

where *δm*, *A*, and *δt* are change of weight (g) after 72 h, over area (m^2^) and test time (h), respectively. The water vapour permeability was reported as the mean (95% CI) of three measurements for both PVA and PVA-PAA samples. The density of samples (PVA and PVA-PAA) was measured by using buoyancy technique utilizing absolute ethanol (ρ = 789 kg·m^−3^). Samples were weighed in air and ethanol and their densities were measured by Equation (3):

(3) $$\rho =\rho_{e}\frac{m_{d}}{m_{d}-m_{e}},$$

where *m*_d_ and ρ_e_ indicate the weight of sample in air and density of ethanol, respectively, and *m*_e_ is the apparent immersed weight.

Scanning electron microscopy (SEM, TESCAN, Brno, Czech) was used to study the surface morphology of dry PVA and PVA-PAA samples and after immersion in solution with pH values of 5, 7, and 10. All samples were vacuum-dried at −80 kPa at 50 °C overnight (LabTech, Bassendean, Australia) prior to sputter coating with platinum.

The bio-adhesion strength of PVA and PVA-PAA samples (*L* × *W* = 3 cm × 3 cm) against pig ear skin obtained from an abattoir was measured using a texture analyser (stable micro systems, Surrey, UK). PVA-PAA samples were equilibrated in different buffers with pH values of 5, 7, and 10, and PVA was equilibrated in distilled water for 24 h before testing. The skin with diameter of ~6 cm was attached with super glue on the surface of a poly methyl methacrylate (PMMA) sheet secured by clamps. The PAA side of the PVA-PAA composite was in contact with skin without application of loading. The edge of the sample was secured in a peeling rig and adhesion testing was performed at the speed of 1 mm/s for each sample until complete detachment occurred. Results were reported as the mean (95% CI) of three replicates.

Tensile tests were performed using a universal testing machine (Instron, Norwood, MA, USA) at a strain rate of 1 mm/s at room temperature until failure. Rectangular samples (*L* × *W* = 5 cm × 1 cm) were kept in a sealed chamber in 65% relative humidity for 48 h before failure testing and were secured by sandpaper and super glue at both ends during testing. Sample thickness was measured using a digital calliper at five different points before testing, and the mean value was used for stress calculation. All mechanical tests were the mean (95% CI) of at least three measurements.

**Figure 2:**
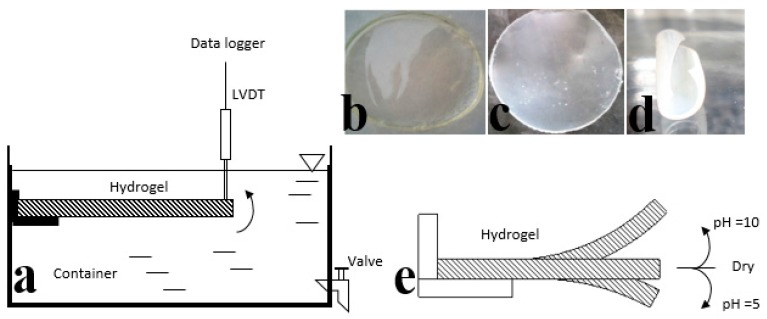
(**a**) Schematic drawing for assessment of pH sensitivity of PVA-PAA samples (the PVA side was upward) using a linear variable displacement transformer (LVDT) sensor. Images of PAV-PAA samples (**b**) Swollen at pH = 5; (**c**) Dry; (**d**) Swollen at pH = 10; and (**e**) Schematic drawing of PVA-PAA samples bending at different pH values.

Deformation of the PVA-PAA hydrogel at different pH values of 5 and 10 was measured using a linear variable displacement transformer (LVDT) sensor, as shown in Figure 2. The cantilever hydrogel films (*L* × *W* = 3 cm × 1 cm) were secured at one end using a cyanoacrylate glue, while the other end was in contact with the sensor. Frequent changes in the pH of swelling media (200 mL) were manually executed using a valve. The vertical movement (mm) of the free end of the hydrogel film was measured by the calibrated LVDT sensor with reference to the position of dry hydrogel at the beginning of the test. The measurement was performed across sequential changes in pH value from 10 to 5.

Cell viability and proliferation were measured for PVA and PVA-PAA samples. The samples were UV sterilised for 20 min and then immersed in DMEM with 1% penicillin streptomycin (Aldrich (St. Louis, MO, USA) and 10% bovine serum and incubated at 37 °C. The extracted solutions of the immersed samples were diluted by the DMEM medium. Human fibroblast cells (each well containing 10^4^ cells) were incubated for 24 h in a 96-well plate. The extract solutions were then added to the plate and incubated under cell culture conditions (5% CO_2_, 20% O_2_, 95% humidity, and 37 °C). After 24 h, 25 μL MTT solution was added to each well and incubation was continued for 4 h, during which dark blue formazan crystals were formed. The optical density of the crystal solutions in 250 μL DMSO was measured at 595 nm wavelength (Eppendorf, Hamburg, Germany) for cell viability assessment. 

Tetracycline hydrochloride was dissolved in water (1 mg/mL) and mixed with PVA solution at room temperature for 15 min, while the double-layered hydrogel was fabricated as described. In vitro release of tetracycline hydrochloride was measured at pH values of 7 and 8 using Franz diffusion cell method, where the pig ear skin was used as the separator membrane. The PAA side of the double-layered composite was in contact with the skin when placed in a donor compartment, and the receptor compartment was filled with buffers at different pH values. The amount of tetracycline hydrochloride release, diffused across the skin, was measured by taking 2 mL aliquots from the receptor solution, replacing it with buffer using a UV spectrophotometer (Eppendorf, Hamburg, Germany) at the wavelength of 363 nm. The antibacterial activity of the antibiotic release against *E. coli* and *S. aureus* was studied using the disc diffusion method. Hydrogel samples were sterilised first with the UV light and placed in *E. coli* (gram negative) and *S. aureus* (gram positive) cultured agar plates and incubated at 37 °C. The diameters of the inhibition zones that developed around the samples were reported at different time points of incubation. 

An independent samples *t*-test was conducted (IBM SPSS Statistics for Windows, Version 22.0. IBM Corp., Armonk, NY, USA), having different test variables using an alpha of 0.05.

## 3. Results 

### 3.1. Swelling Behaviours

**Figure 3:**
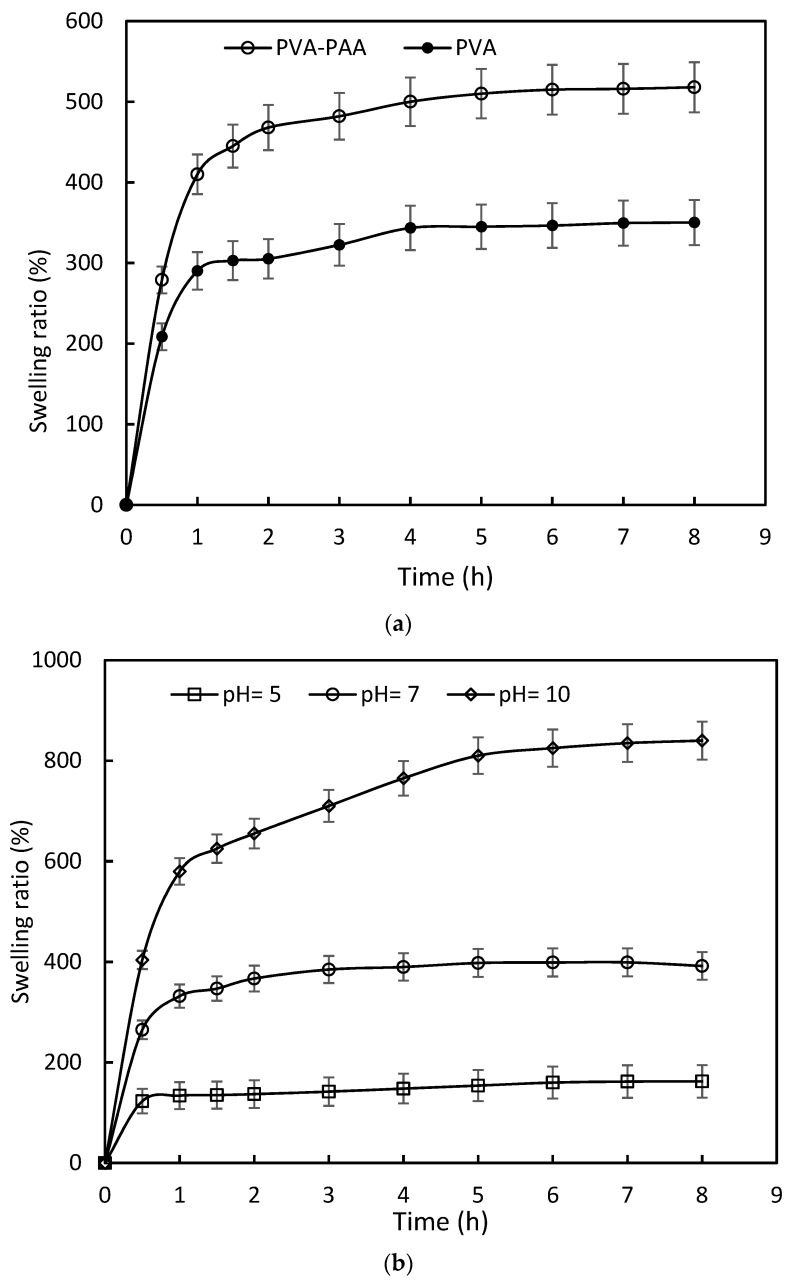
(**a**) Swelling properties of PVA-PAA and PVA samples in distilled water; and (**b**) Kinetics of swelling for PVA-PAA samples at different pH values.

Addition of the PAA layer to the PVA hydrogel significantly affected the equilibrium swelling ratio (*p* = 0.035) of double-layered hydrogel compared to PVA, as shown in Figure 3a. In contrast to PVA, the PVA-PAA hydrogel was sensitive to pH change, as shown in Figure 3b. The equilibrium swelling ratio of the PVA-PAA hydrogel increased by 48% compared to that of PVA in distilled water. At different pH values of 5 and 10, the equilibrium swelling ratio was altered by 115% and −58%, respectively, relative to that of at pH = 7 (*p* < 0.02). The equilibrium swelling time was measured as 7, 3, and 1 h for the PVA-PAA hydrogel at different pH values of 10, 7, and 5, respectively, as shown in Figure 3b.

### 3.2. Surface and Morphological Studies

In Table 1, it can be seen that the PVA-PAA hydrogel displayed a lower contact angle, while its water vapour permeability significantly decreased (*p* = 0.04) compared to that of the PVA hydrogel. The findings of higher surface hydrophilicity and lower water vapour permeability were consistent with the observations of increased swelling ratios of the PVA-PAA hydrogel. 

**Figure 4:**
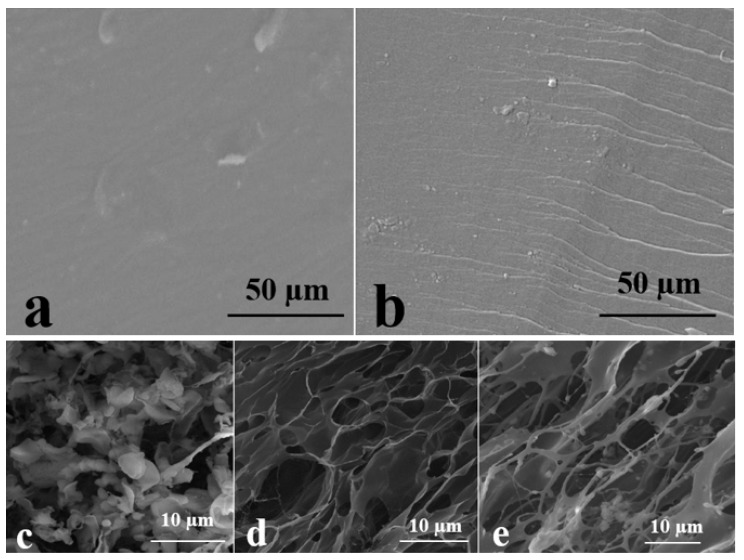
SEM images of dry (**a**) PVA and (**b**) PVA-PAA samples and the surface morphology of PVA-PAA samples at different pH values of (**c**) 5; (**d**) 7; and (**e**) 10.

Different surface morphologies of PVA and PVA-PAA hydrogels were observed from SEM, as shown in Figure 4a,b, indicating that the smooth surface of the PVA changed after polymerisation of the AA monomer and deposition of the PAA layer. The SEM images also revealed that changes in surface porosity occurred after exposure of hydrogel to the solution with different pH values, as shown in Figure 4c–e. Smaller pores and denser hydrogel networks were found at pH = 5 (Figure 4c) compared to samples swollen at pH = 7 (Figure 4b). The increase of pH to 10 resulted in larger porosity and lower hydrogel density (as shown in Figure 4e), while substantial increases in both number and size of porosities were observed. These SEM results were consistent with the results of swelling kinetics analysis and swelling ratio measurement, where a higher swelling ratio equilibrium occurred at pH = 10. 

### 3.3. Spectroscopic Study

**Figure 5:**
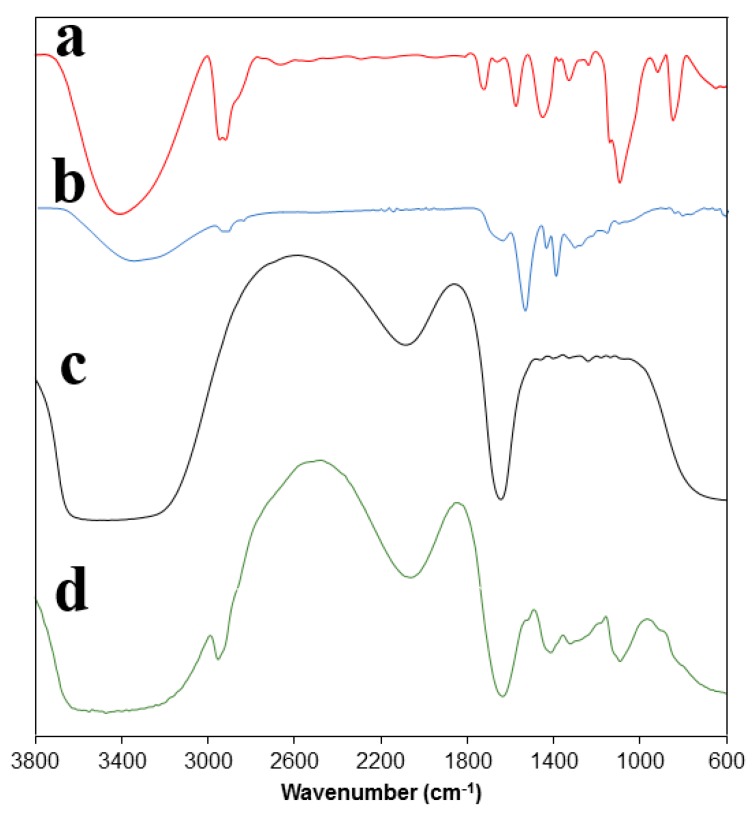
Fourier Transform Infrared (FTIR) spectra of (**a**) PVA; (**b**) PVA-PAA; (**c**) Tetracycline; and (**d**) PVA-tetracycline.

FTIR spectra confirmed the deposition of PAA hydrogel on the PVA hydrogel surface, as shown in Figure 5. The intensity of the peak at 1760–1730 cm^−1^ was weak in the PVA hydrogel, indicating a low concentration of acetate groups. Additionally, a broad C-H alkyl stretching band at 2850–3000 cm^−1^ and a strong hydroxyl band for non-bonded stretching band at 3600 cm^−1^ were present in the PVA hydrogel. A peak was verified at 1105 cm^−1^ which reflected the semicrystalline structure of the PVA, as shown in Figure 5a. After PAA deposition, the FTIR spectrum was different from what was observed for the PVA. It showed the stretching of C=O and stretching vibrations of OH groups at 3390–3180 cm^−1^ and 1690 cm^−1^, respectively. The peaks at 1510 and 1405 cm^−1^—associated with scissoring and vibrations of –CH_2_– and CH–CO groups, respectively—are shown in Figure 5b. The FTIR spectrum of tetracycline showed a strong peak at 3450–3180 cm^−1^ representing N–H and O–H and aromatic C–H stretching bonds. The band of medium intensity observed at a frequency of 2130 cm^−1^ was assigned to symmetrical stretching vibration of the methyl group. The vibrational peak at 1648 cm^−1^ was assigned to C=C stretching. Aromatic in-plane and out-of-plane deformation peaks appeared at 1247–1000 cm^−1^ and 707–501 cm^−1^, respectively, as shown in Figure 5c. The FTIR data of different samples were well supported by reported data for PVA [34,35], PAA [36], and tetracycline [37]. 

### 3.4. Mechanical Properties

**Figure 6:**
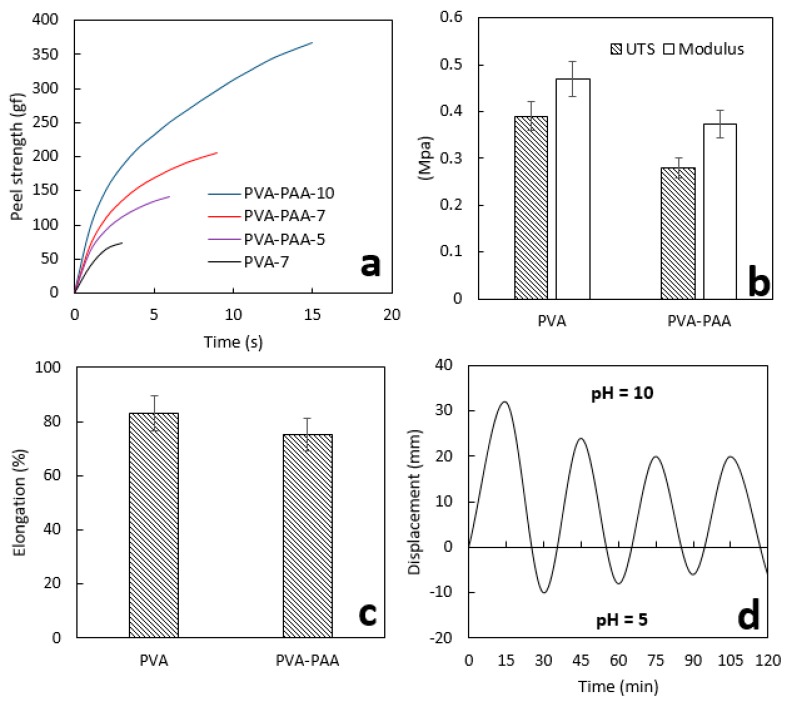
(**a**) Adhesion and (**b**,**c**) mechanical properties of PVA and PVA-PAA hydrogels and (**d**) pH sensitivity results of PVA-PAA samples (the PAA side was upward) at different pH values of 5 and 10. UTS: ultimate tensile strength.

The PVA-PAA double-layered hydrogel was found to be more adhesive than the PVA at pH = 7, with improvements in adhesion force and time of 170% and 200%, respectively, as shown in Figure 6a. The maximum adhesion force was measured as 140, 205, and 370 gf (gram force) for the PVA-PAA hydrogel at pH = 5, 7, and 10, respectively. An increase in adhesion time from 6 to 8.5 and 15 s was observed with pH values increasing from 5 to 7 and 10, respectively. Not surprisingly, the bio-adhesion property of the swollen PVA-PAA hydrogel at pH = 5 was higher than that of the PVA at pH = 7, since the PAA hydrogel was able to produce stronger adherence via the creation of more secondary forces (e.g., van der Waals, hydrogen bonding, and electron transfer), compared to the PVA hydrogel. No mechanical rupture or failure was observed for all samples during the adhesion tests. 

Statistical analysis revealed that increasing the pH values from 7 to 10 for the PVA-PAA hydrogel as well as the addition of PAA to the PVA hydrogel at pH = 7 significantly increased adhesion forces (*p* < 0.04). No significant difference in adhesion forces for PVA-PAA was found between pH = 5 and 7, as shown in Figure 6a. No significant difference was found for ultimate tensile strength (UTS), modulus (Figure 6b), or elongation at break (Figure 6c) between PVA and PVA-PAA hydrogels. Thus, surface modification of the PVA hydrogel by the addition of PAA did not significantly affect the bulk mechanical properties. The PVA-PAA hydrogel was found to preserve its pH sensitivity after four cycles of swelling at different pH values (as shown in Figure 6d), while convex and concave deformation were observed at pH = 5 and 10, respectively. The maximum stable displacement for free ends of the hydrogel was measured as 20 and −5 mm at pH = 10 and 5, respectively. The swelling capability of the PVA was independent of pH value, while the PAA swelled to a level similar to that of the PVA at pH = 10 and 5, respectively. Therefore, the different swelling properties are responsible for the observed positive or negative bending deformations. 

### 3.5. Biocompatibility

**Figure 7:**
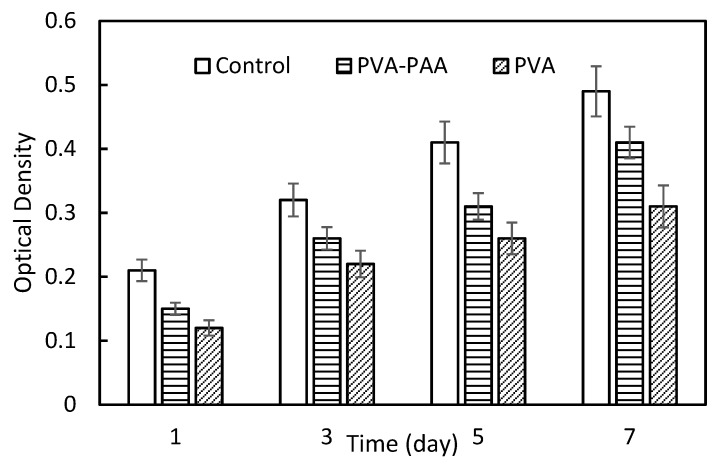
Biocompatibility analysis for PVA and PVA-PAA samples.

The MTT assay was conducted to evaluate the cell viability and quantify the rate of cell proliferation in PVA-PAA samples and to compare them with the results from the PVA and control. As an index for cell viability, the optical density was seen to increase with time for both PVA and PVA-PAA hydrogels. With respect to the higher hydrophilic property of the PVA-PAA hydrogel which was observed during measurement of the contact angle and swelling ratio, increments of optical density from day 1 to 7 were observed, indicating its appropriate biocompatibility. Significant increases in optical density of PVA and PVA-PAA were seen on day 7 (*p* = 0.016), as shown in Figure 7. 

**Figure 8:**
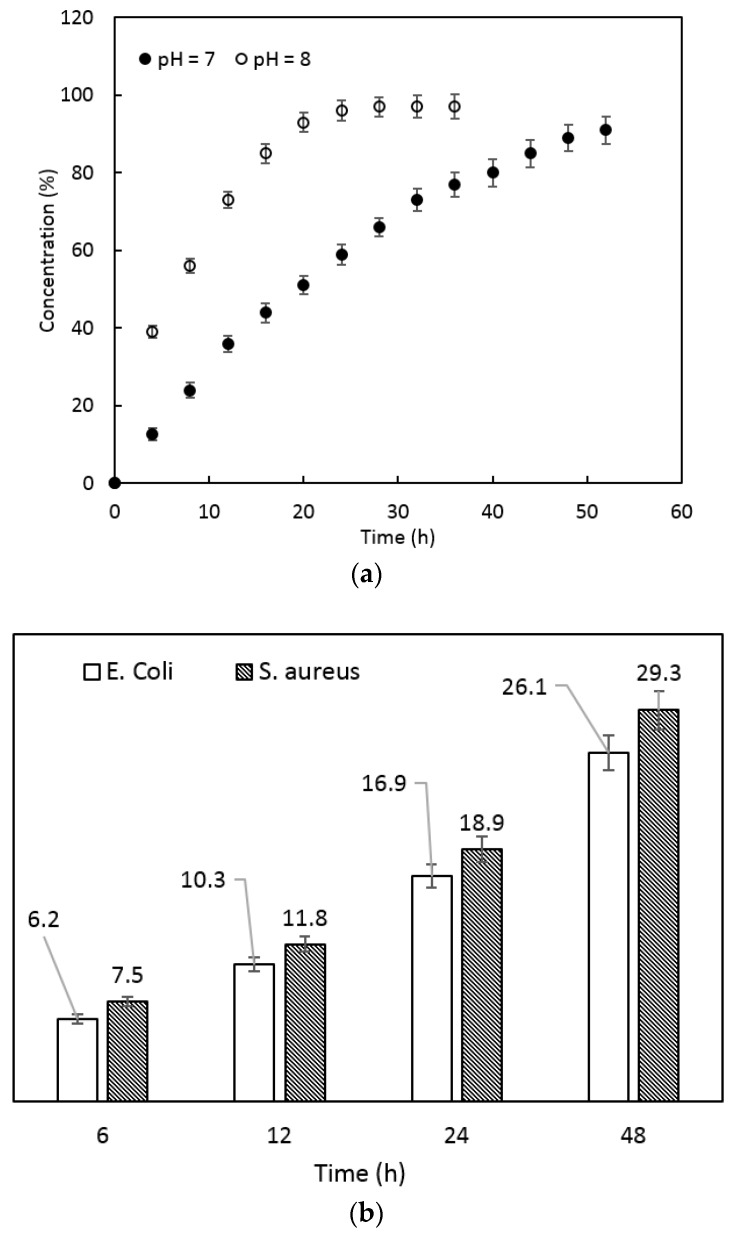
(**a**) Tetracycline release from PVA-PAA samples at different pH values and (**b**) Changes in the diameter of the inhibition zone at different time points.

Tetracycline hydrochloride was used in this study, and acted as the broad-spectrum bacteriostatic agent for the treatment and prevention of bacterial skin infections. Tetracycline is effective against infections caused by *Trachoma, Rickettsiae*, *Mycoplasma* and *Chlamydia*, as well as gram positive and gram negative bacteria. An increase of the pH values of swelling media from neutral to 8 was shown to affect the release profile of tetracycline over time, as shown in Figure 8a. At pH = 7, the PVA-PAA hydrogel could deliver sustained release of antibiotic for approximately 50 h, whereas an increment in pH resulted in a faster release. In a base with pH = 8, a rapid initial rate of antibiotic release was observed, and the entire antibiotic was released in less than 20 h. Sensitivity of the PVA-PAA wound dressing to pH provides the opportunity for controlling wound bed infection, since a higher amount of antibiotic release was seen with an increase in the pH value, which reflected infected wounds. In fact, wound infection would encourage the PVA-PAA wound dressing to release a higher concentration of antibiotics over a shorter period, leading to superior wound healing management and infection control. Increases of the diameter of the inhibition zone as a function of time were seen for both *E. coli* and *S. aureus* bacteria, while unsurprisingly there was no significant difference in the measured diameter between gram positive and negative bacteria at any time point, as shown in Figure 8b. Observation of changes in the diameter of the inhibition zone around samples at different time points seemed to be consistent with the release kinetics profiles of the antibiotics. The inhibition zone reached its maximum diameter after 48 h, where the relevant concentration of antibiotic from sample was at its maximum. 

## 4. Discussion

In this study, after achieving a cost-effective and simple method for the fabrication of a double-layered hydrophilic structure, a solution to the previous difficulties regarding the stability of layers is addressed. Previous studies of the use of oppositely-charged gels for the construction of multilayer films reported high sensitivity to environmental conditions, where stability could be affected by temperature, ionic strength, charge density, and pH ranges [38,39,40,41,42]. However, the impact of external and compositional parameters on the stability of layers was not seen with our method of preparation. The high stability of the double-layered hydrogel composite—as demonstrated in swelling process, mechanical properties, and pH sensitivity analysis—is likely to reflect the physical entanglement and possible formation of hydrogen bonds at the interface. Since the polymerization process of acrylic acid monomer was more likely to start after penetration into the swollen PVA, a physical entanglement between PVA and PAA chains could occur. Moreover, the existence of OH and COOH groups provided the opportunity to form hydrogen bondings, effectively increasing the interface stability. It seems that the combination of hydrogen bonds formation, together with the existence of physical entanglement of PVA and PAA chains improved the interface, mechanical, and biomedical properties of the composite. Based on that method, we found significant differences in selected properties between PVA and PVA-PAA wound dressings. Swelling ratio, contact angle, biocompatibility, adhesion property, and water vapour permeability were significantly improved in the double-layered hydrogel compared to the values with the PVA monolayer. With the trend of higher hydrophilicity properties, the mechanical properties of the PVA-PAA hydrogel—including ultimate tensile strength, modulus, and elongation at break—remained approximately unchanged compared to those of PVA. Release characterisation of the PVA-PAA wound dressing at different pH values revealed a significantly higher release rate at pH = 8 than at pH = 7. Meanwhile, the hydrophilic property of the PVA-PAA wound dressing was increased compared to that of the PVA; the effect of the higher swelling capability of the PAA (compared to the PVA) on the PVA-PAA hydrogel resulted in a positive or negative bending deformation. This deformation could potentially be used as an indicator to identify an infected wound bed, since the occurrence of infection is associated with an increase in the pH value [6]. Moreover, the higher rate of antibiotic release after an increase in the pH value in the wound bed is an efficient mechanism for infection control. The increase in the pH value increased the rate of carboxyl group dissociation, resulting in a higher accumulation of negative charges within the PAA hydrogel. Repulsion between the fixed negative charges increased the pore size, enhanced water uptake capability, and increased the rate of antibiotic release.

On the basis of the measured mechanical properties, it is likely that the PVA layer was more responsible for load transfer than the PAA layer—a finding which is consistent with other studies showing that the mechanical properties of the PAA were weak [17,43]. The PVA hydrogels with a high degree of hydrolysis (>99.9%) exhibited acceptable biomechanical properties, while available hydroxyl groups provided a high density of intra-molecular hydrogen bonding and formed larger and more crystalline regions compared to PVA hydrogels with a lower degree of hydrolysis. The formation of folded chains in crystalline regions increased the physical entanglement of molecules, resulting in enhanced mechanical properties. Measurement of the degree of crystallisation (X) based on density (Equations (4) and (5)) revealed an approximately similar crystallisation degree before and after PVA surface modification [44].

(4) $$\frac{1}{\rho_{g}}=\frac{X}{\rho_{c}}+\frac{1-X}{\rho_{a}},$$

(5) $$\frac{1}{\rho_{h}}=\frac{1-W_{\mathrm{PVA}}}{\rho_{H2O}}+\frac{W_{\mathrm{PVA}}}{\rho_{g}},$$

where ρ_c_ = 1.345 g·cm^−3^ and ρ_a_= 1.269 g·cm^−3^ were the densities of 100% crystalline and amorphous PVA, respectively [44,45]. ρ_g_, ρ_h_, and ρ_H2O_ were the densities of PVA at dry, PVA with 65% relative humidity, and water, respectively. *W*_PVA_ was the weight percentage of the PVA hydrogel. The finding of negligible change in the crystallinity density of samples (X), due to similar densities for PVA and PVA-PAA samples (*W*_PVA_ and *W*_PVA-PAA_) at 65% relative humidity, was consistent with mechanical results that identified non-significant differences in mechanical properties.

The increases in adhesion force and time are consistent with the concept of the diffusion theory and the adsorption mechanism, where interpenetration of the rough surface of the wound bed and formation of secondary bonding were known to be responsible for adhesion. Therefore, the larger pore size and higher porosities which appeared at a higher pH value led to the enhanced adhesion force and time. The increase in the wettability of the PVA surface by the addition of PAA hydrogel (Table 1)—a factor that resulted in the formation of a closer contact between skin and hydrogel on a molecular scale—was therefore a primary reason for the observed enhancement of the bio-adhesion property. On the basis of the adsorption theory, upon the formation of a smoother contact interface, it was likely that specific types of bonding including London, Debye, or Keesom forces were present [46]. Moreover, it was apparent that increases in the dimension and number of porosities played a role in enhancing the bio-adhesion properties of the PVA-PAA hydrogel and could increase the likelihood of penetration of skin into the hydrogel pores (Figure 4). With reference to diffusion theory, although skin and hydrogels may exhibit macroscopic thermodynamic incompatibility, some inter-diffusion can be accepted between them [47]. An appropriate rate of water loss which can control wound bed moisture and prevent excessive dehydration is an important factor that reduces the risk of wound bacterial infection while enhancing the healing process. As observed in our study, the higher water uptake capacity provided by the PAA layer introduced a physical barrier against wound bed dehydration. While the current study revealed that the proposed simple method for fabrication resulted in the introduction of a double-layered hydrogel with enhanced biomedical properties, more studies are required to characterize the interfacial properties of the composite. 

## 5. Conclusions

The current study introduced a simple method for the fabrication of a PVA-PAA double-layer composite. The results of this study revealed that polymerisation of the AA monomer on the surface of the partially swollen PVA resulted in the fabrication of a double-layered hydrogel with enhanced biomedical properties. Additionally, the addition of PAA led to improved swelling, adhesion, and biocompatibility properties, while the mechanical properties of the PVA remained approximately unchanged. Furthermore, the PVA-PAA hydrogel exhibited a degree of sensitivity to pH change, making it suitable for a variety of biomedical applications.

## Figures and Tables

**Table 1:** Change in contact angle and permeability, mean (95% CI), of PVA and PVA-PAA samples.

Hydrogel	Contact Angle (°)	Water Vapour Permeability (g·m^−2^·day^−1^)
PVA	33.3 (2.3)	1631.5 (12.3)
PVA-PAA	24.2 (1.2)	1209.1 (25.2)
